# Applying systematic review search methods to the grey literature: a case study examining guidelines for school-based breakfast programs in Canada

**DOI:** 10.1186/s13643-015-0125-0

**Published:** 2015-10-22

**Authors:** Katelyn Godin, Jackie Stapleton, Sharon I. Kirkpatrick, Rhona M. Hanning, Scott T. Leatherdale

**Affiliations:** School of Public Health and Health Systems, University of Waterloo, 200 University Avenue West, Waterloo, ON N2L 3G1 Canada

## Abstract

**Background:**

Grey literature is an important source of information for large-scale review syntheses. However, there are many characteristics of grey literature that make it difficult to search systematically. Further, there is no ‘gold standard’ for rigorous systematic grey literature search methods and few resources on how to conduct this type of search. This paper describes systematic review search methods that were developed and applied to complete a case study systematic review of grey literature that examined guidelines for school-based breakfast programs in Canada.

**Methods:**

A grey literature search plan was developed to incorporate four different searching strategies: (1) grey literature databases, (2) customized Google search engines, (3) targeted websites, and (4) consultation with contact experts. These complementary strategies were used to minimize the risk of omitting relevant sources. Since abstracts are often unavailable in grey literature documents, items’ abstracts, executive summaries, or table of contents (whichever was available) were screened. Screening of publications’ full-text followed. Data were extracted on the organization, year published, who they were developed by, intended audience, goal/objectives of document, sources of evidence/resources cited, meals mentioned in the guidelines, and recommendations for program delivery.

**Results:**

The search strategies for identifying and screening publications for inclusion in the case study review was found to be manageable, comprehensive, and intuitive when applied in practice. The four search strategies of the grey literature search plan yielded 302 potentially relevant items for screening. Following the screening process, 15 publications that met all eligibility criteria remained and were included in the case study systematic review. The high-level findings of the case study systematic review are briefly described.

**Conclusions:**

This article demonstrated a feasible and seemingly robust method for applying systematic search strategies to identify web-based resources in the grey literature. The search strategy we developed and tested is amenable to adaptation to identify other types of grey literature from other disciplines and answering a wide range of research questions. This method should be further adapted and tested in future research syntheses.

**Electronic supplementary material:**

The online version of this article (doi:10.1186/s13643-015-0125-0) contains supplementary material, which is available to authorized users.

## Background

Grey literature is an extensive, though complex, source of information. The ‘Luxembourg definition’ offers a widely accepted description for grey literature as ‘that which is produced on all levels of government, academics, business and industry in print and electronic formats, but which is not controlled by commercial publishers, i.e., where publishing is not the primary activity of the producing body’ [1]. These documents are not formally published in academic sources (i.e. books or journals) and include items such as reports, theses, conference proceedings, newspapers, fact sheets, websites, and policy documents. In addition, unpublished research and data may be considered forms of grey literature [2]. The Internet is often used as a platform for publishing grey literature by a wide range of organizations, such as government and non-government organizations, research centres, health institutes, and non-profit organizations, contributing to a proliferation of this source of data [3]. Grey literature documents can serve as valuable resources for practitioners and decision-makers across disciplines, since these documents often contain policy- and research-relevant information (e.g. clinical practice guidelines, research reports, program evaluation studies, legislation) from authoritative sources and tend to be widely accessible (i.e. subscriptions are not required as may be the case with the peer-reviewed scholarly literature).

To achieve a comprehensive review, grey literature is an important element of large-scale syntheses and can be incorporated in two ways: (1) as included items in these reviews and (2) as a means to identify relevant studies and publications for these projects. The Cochrane Handbook for Systematic Reviews of Interventions and the Institute of Medicine Standards for Systematic Review recommend incorporating grey literature in systematic reviews [2, 4]. For reviews of intervention studies, failure to include grey literature may artificially amplify estimates of treatment effects, given the effects of publication bias [5]. Further, there is often a considerable lag in the period between research and publication and sometimes, research is never formally published. Relying exclusively on peer-reviewed literature could thus omit potentially relevant work [6]. Applying systematic methods to grey literature searches should improve the quality of review syntheses that include grey literature by providing a more comprehensive and less biassed set of reports to examine.

In general, systematic review search methods should be explicit, reproducible, and attempt to identify all documents that meet the pre-defined eligibility criteria for inclusion in the review [2]. These standards can theoretically be applied to search methods used to capture grey literature. However, grey literature searches are often not as systematic as traditional systematic searches of academic literature [7]. Accessing material published on the Internet represents a challenge for the systematic reviewer, given the vast amount of information, lack of standard indexing and controlled vocabulary, and lack of archiving. The coverage of grey literature is patchy across mainstream databases. Conference proceedings and dissertations are found in some databases (e.g. Embase and Web of Science), but other forms of grey literature (e.g. industry and government reports) are rarely found in mainstream databases. Several researchers have outlined various search methods for grey literature searches identifying web-based resources [3, 8–10]. However, there is no ‘gold standard’ for rigorous systematic grey literature search methods and there are few resources on how to conduct this type of search. For example, the Cochrane Handbook, often cited as the gold standard for conducting systematic reviews, provides limited guidance and specificity for grey literature search methods [2]. In addition, the reporting of grey literature search methods in systematic reviews are often not held to the same high standards in transparency and reproducibility as the academic database search methods [11].

This paper describes systematic review search methods that were developed and applied to conduct a systematic review of grey literature pertinent to guidelines for school-based breakfast programs in Canada. This review represents a suitable case study of grey literature search methods for two reasons. First, guidelines for such programs are typically released by government and non-government organizations and not published in academic journals. Second, this topic area warrants investigation, as breakfast programs have recently been featured extensively in the political and popular media. The physical, social, and cognitive benefits of eating breakfast are well-established [12–14], yet Canadian studies demonstrate a high prevalence of breakfast skipping among youth [15, 16]. School breakfast programs in Canada are widespread and growing in number [17]. However, student-level participation in these programs is often very low [15, 18]. While there is some evidence that these programs can yield similar benefits to those witnessed in other countries [19, 20], there have been few robust evaluations of these programs and little evidence of ‘what works’ in the Canadian context [17, 20]. The disparity between the availability of breakfast programs and low uptake by youth may suggest that Canadian schools may lack guidance on how to effectively deliver these programs to a wide demographic. Further, it is unclear what ‘best practices’ or other evidence is incorporated into the guidelines for these programs. As such, the goal of the systematic review was to identify guidelines in the grey literature for school-based breakfast programs in Canada and examine the common and conflicting recommendations and sources of evidence within these guidelines.

The objectives of this paper are to describe a template that was developed and applied to conduct a systematic search of the grey literature (focusing primarily on online resources) and to illustrate the type of records that can be identified using these methods. The focus of the paper is to demonstrate how the systematic search methods can be applied to the grey literature in practice. Other review components beyond search methods are also addressed, including eligibility criteria, publication selection, data collection processes, and reporting standards. The key findings of the review are briefly highlighted.

## Methods

Prior to conducting the grey literature search, it is essential to develop a detailed search plan which forms the search methods section of the systematic review protocol [2]. A grey literature search plan should outline the resources, search terms, websites, and limits to be used, prior to conducting the search [2]. The methodological plan provides guidance, structure, and transparency to the search methods, ensuring comprehensive and organized search methods, and reduces the risk of introducing bias into the search methods. Developing a search method plan is also important for time management, as it sets boundaries for the number of search terms and volume of results to be screened. Creating a plan and documenting each step throughout the search process helps to ensure compliance with systematic review reporting standards, such as PRISMA (Preferred Reporting Items for Systematic Reviews and Meta-Analyses) [21]. Elements to be reported, according to PRISMA, include a description of all information sources in the search, the name of the person conducting the search, the date the search was performed, and a full search strategy of at least one database including all search terms and combinations. These reporting standards should be applied to a grey literature search.

### Eligibility criteria

The case study used for the development of the systematic grey literature search and review examined guidelines for the delivery of breakfast programs in Canadian schools. The review’s eligibility criteria were defined in the grey literature search plan and are shown in Table 1. These criteria demonstrate that the usual method of systematically searching academic journal databases is inappropriate for adequately addressing the case study review’s research questions.

**Table 1:** Case study review eligibility criteria

Inclusion criteria	Exclusion criteria
Published by a government or NGO at either the federal or provincial/territorial (P/T) level within Canada	Published outside of Canada
	Published by an organization at the school board, public health unit, municipal, or regional levels
Available in English	Unavailable in English
Most current version of the document	Document was a draft or summary version or has been replaced with another document
Intended for school administrators/meal program coordinator at Canadian elementary or secondary schools	Primarily intended for a student or parent/guardian audiences
Included standards or guidelines for school-based breakfast programs in at least two of the following areas: food safety, nutrition, program delivery, or community partnerships	Did not contain considerations for breakfast programs
	Described breakfast events/programs in daycare or community settings
	Only described nutrition guidelines for schools without specific breakfast program-related considerations were excluded
	Merely provided resources from external groups as an appendix
Breakfast program was described as any program that involves the provision of breakfast or a mid-morning snack to students in a school setting or nearby (e.g. neighbouring church or community centre) for students at little to no cost	Newsletters, news releases, or memorandums

### Information sources and searching strategies

A grey literature search plan was developed to incorporate four different searching strategies: (1) grey literature databases, (2) customized Google search engines, (3) targeted websites, and (4) consultation with contact experts. These strategies were loosely adapted from those used in a grey literature review on drug prevention initiatives [22]. In contrast to the earlier review, the strategies presented here are more distinct from each other and described in greater detail, allowing them to be reproduced in future research. Several complementary strategies to identify publications were employed to minimize the risk of omitting relevant sources. For example, Avenell and colleagues found that relying on a single database as an information source for their review on nutritional supplementation trials would have omitted nearly half of all eligible documents [23]. Since databases and search engines use unique algorithms to generate their relevance rating schemes, using a variety of these information sources is likely to lead to a broader reach of records. As with any synthesis, documentation of each stage of the grey literature search process is extremely important to demonstrate transparency and comprehensiveness. Thus, all assumptions, decisions, and challenges throughout the review were recorded.

The first searching strategy involved searching grey literature databases relevant to the subject of the review. Databases that catalogue grey literature documents provide indexing and in some cases, a level of peer review for both print and online resources. However, these databases can have a wide variance in search functionalities and filters available for retrieving results (i.e. ability to search by title and abstract). As such, researchers must adapt their search terms to fit the databases they use in their search. In this case study, a search was conducted on March 20, 2015 and included three databases: Canadian Research Index (ProQuest, Micromedia), the Canadian Electronic Library—Canadian Public Policy Collection, and the Canadian Electronic Library—Canadian Health Research Collection. The search strategy included the following three groups of terms: (1) school; (2) breakfast (e.g. meal, nutrition, breakfast, snack, milk, feeding, lunch); and (3) guidelines (e.g. guidelines, standards, frameworks, recommendations, best practices). The terms were used as keyword fields in all database searches. The results of the database searches were exported to an Excel spreadsheet, and duplicates were excluded through the ‘remove duplicates’ function. The titles of all search ‘hits’ were reviewed in Excel, a step that is analogous to a title screen in a traditional review of peer-reviewed academic articles. Titles that appeared relevant were highlighted in Excel and retained for further screening.

The second search strategy involved conducting Google searches for documents published on the Internet. Searching Google can be overwhelming due to the vast amount of information and lack of consistent organization across websites. A typical systematic search strategy for an academic database includes one search strategy combining all search terms for which all results will be screened for eligibility [2]. In contrast, Google searches may require creating several search strategies containing multiple combinations of search terms. Also, it is impossible to screen all retrieved results from Google searches. Instead, one must rely on the power of relevancy ranking within Google search engines to bring the most relevant results to the top of the list and set the number of pages to be screened in advance to ensure consistency across searches and effective time management. Fortunately, custom Google search engines have been developed to narrow the search results to a specific subject area and/or website, allowing more refined and targeted searching.

Specifically, for the second search strategy, custom Google search engines for Canadian public health information and government documents were utilized. The custom search engine for Canadian public health information was created by the Ontario Public Health Libraries Association and captures the websites of Canada’s federal and provincial health departments, public health agencies, and collaborating centres [24]. The custom search engine for government documents was available courtesy of MADGIC, Carleton University and hosted by the University of Waterloo [25]. This custom search engine captures government publications from federal, provincial, and local government bodies, and was filtered to only capture Canadian government documents. Ten unique search strategies were applied to each search engine, as shown in Additional file 1. The first ten pages of each search’s hits (representing 100 results) were reviewed, using the title and short text underneath. This number of pages was chosen to capture many of the most relevant hits while still being a feasible amount to screen. Potentially relevant records were ‘bookmarked’ in the web browser used at the time of searching (Google Chrome) and later entering each into an Excel spreadsheet. Each bookmarked homepage was filed into a sub-folder that was named after the specific search strategy by which it was identified, within a main folder that was named after the search engine used. Filing bookmarks this way enabled the reviewers to access the bookmarked websites via the browser’s Bookmark Manager and see which websites were identified through which search terms and engine. Bookmarking potentially relevant articles at the time of screening also prevented the same record from being identified repeatedly throughout this search strategy, since the URL of previously bookmarked pages are starred, therefore easily identifiable when viewing the page. This feature allowed us to easily track new records identified through each search. Titles that were identified as potentially relevant were retained for further screening. For each search strategy, the search terms and the number of results retrieved and/or screened were recorded.

The third search strategy involved browsing targeted websites of relevant health organizations and agencies, similar to the hand-searching methods used in screening a journal’s table of contents in systematic review search methods. The identification and selection of relevant organizations for a grey literature search can include soliciting recommendations from content experts on the research team as well as reviewing published lists of organizations relevant to the research area. One such useful publication includes ‘Grey Matters – A Practical Deep Web Search Tool for Evidence based Medicine’, by the Canadian Agency for Drugs and Technologies in Health [26]. This checklist of Canadian and international health organizations and agencies is of particular relevance for Canadian health researchers. For this case study, the researchers used Google to identify the organizations publishing material on Canadian breakfast programs. Google can be an efficient tool for locating organizations publishing on very specific topics (such as Canadian breakfast programs) and offers an advantage over published lists of websites which can quickly become out of date as websites change or disappear entirely.

This process was broken down into two steps. First, a Google search was conducted to identify the relevant organizations and websites publishing documents on the relevant subject area. Next, each of the relevant websites’ homepage were ‘hand-searched’ for potentially relevant documents (e.g. web pages, reports). Within this step, each website and the date on which each search was conducted were documented. The targeted web searches took place on April 13, 2015. The same ten unique search strategies used in the custom Google search engine searches were applied to a Google search engine, as shown in Additional file 2. A filter was applied to each search to capture only Canadian websites. The first ten pages of each search’s hits (again, representing 100 results) were reviewed for potentially relevant titles (supplemented with the text under the title). Seventy-seven websites were identified in this step (see Additional file 3). The websites’ name/organization and URL were entered into an Excel spreadsheet. Each of these websites were later searched using the websites’ database or search bar using combinations of keywords. Websites that did not have a database or search bar were hand-searched. Hand-searching the websites’ homepages yielded 126 potentially relevant records. The name, year, and URL of these individual records that appeared relevant through hand-searching were also entered on the Excel spreadsheet within the same row as its main website homepage. Potentially relevant records continued to the next screening phase.

The fourth search technique used to identify documents for screening involved contacting content experts to identify other items for possible inclusion in the review. Individuals that are well-versed in the topic of the research synthesis and likely to be aware of relevant documents were considered content experts. To improve the comprehensiveness of this technique, the content experts contacted were chosen to reflect the diversity of the topic under investigation (i.e. span disciplines and geographical areas), if applicable. The number of content experts contacted may vary depending on the scope of the review and project timelines. Ideally, researchers should attempt to reach data saturation, in which addition of other content experts is unlikely to uncover additional relevant publications. Excel was used to document contact attempts, who had responded, identified documents, and the need for reminder messages to be sent to those who had not yet replied.

In this case study, a wide variety of content experts (*n* = 35, including breakfast program coordinators, dietitians, government representatives, school food caterers, nutrition researchers, program funders) were contacted by email on March 30, 2015. These experts represent a range of stakeholders from all of the Canadian provinces and territories. The email message briefly explained the aims of the project and requested that the recipient identify or send any potentially relevant documents or sources and/or forward the message to colleagues who could potentially provide assistance. If a content expert did not reply to the initial inquiry within a 7-day period, a reminder email was sent. There were no further attempts to contact non-responders beyond this second email. All items identified by the content experts before April 21, 2015 (representing a 3-week window of opportunity) were considered and proceeded for further screening. Twenty nine of the 35 (82.9 %) experts contacted and/or referred by a colleague responded to the email inquiry.

### Eligibility assessment and study selection

**Fig. 1:**
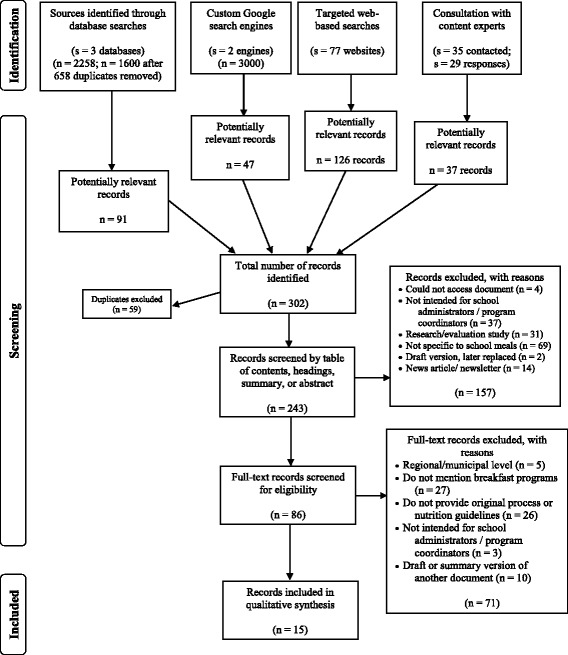
Study flow diagram. This figure depicts the three phases of the review, including the number of records screened and included in the final synthesis

PRISMA recommends using a study flow diagram to describe the screening and study selection process [21]. This process was applied to the grey literature search methods (Fig. 1). The title and source organization of documents identified and deemed relevant from the four search techniques in the case study were entered into an Excel sheet, and duplicates were excluded using Excel’s remove duplicates function. Since abstracts are often unavailable in grey literature documents [3], the abstracts, executive summaries, or tables of contents (whichever were available) of items were reviewed for relevance to the research objectives by one author. If more than one of these elements were available, all were reviewed for relevance. Next, the full-text of all items that moved to the second stage of screening was reviewed. When it was unclear whether or not an item met the eligibility criteria during screening, the reviewer erred on the side of caution and the item continued for further screening. We assigned a numeric code for each reason for excluding items (i.e. ‘1’ for ‘include’, ‘2.1’ for ‘exclude – could not access document’, ‘2.2’ for ‘exclude – not intended for school administrators/program coordinators’). We recorded a screening code for each item at both stages of screening. All items that remained following full-text screening were included in the review.

### Data collection process and synthesis of results

Following full review of each included publication within the case study, data were extracted pertaining to the source organization, year published, by whom they were developed, intended audience, goal/objectives of document, sources of evidence/resources cited, meals mentioned in the guidelines, and recommendations for program delivery. Only data that were relevant to school breakfast programs were extracted (i.e. guidelines pertaining to vending machines or relative pricing of healthy to unhealthy foods were not extracted), consistent with the a priori objectives of the review.

## Results

The four search strategies of the grey literature search plan yielded 302 potentially relevant items for screening (see Fig. 1). Following the screening process, 15 publications that met all eligibility criteria remained and were included in the case study systematic review [27–31]. These publications primarily took the form of reports and websites. Table 2 describes other characteristics of the included publications. Some of the publications did not explicitly state key information, such as date of publication and contributors. In such cases, this information was gathered from other sources, such as the organization’s website and/or publications.

**Table 2:** Document characteristics

Document	Organization, type	Year	Strategies used to identify document	# strategies
Breakfast for learning start-up kit [35]	Breakfast for Learning, national-level NGO	2014	Content experts; targeted web searches	2
Build them up! Guide for schools [36]	Breakfast Club of Canada, national-level NGO	2015	Content experts; targeted web searches	2
Cram nutrition program resources [33]	Child Nutrition Council of Manitoba, P/T level NGO	2012	Targeted web searches	1
Who’s hungry? How to start a breakfast program at your school [32]	New Brunswick Teachers’ Association, P/T level NGO	2012	Content experts	1
Nourish Nova Scotia resources [37]	NNS, P/T level NGO	2015	Content experts; targeted web searches	2
Healthy eating guidelines for school nutrition programs: a resource for first nations schools [38]	Cancer Care Ontario, P/T level NGO	2012	Custom Google search engines; targeted web searches	2
Nutrition tools for schools resources [39]	Ontario Society of Nutrition Professionals in Public Health, P/T level NGO	2015	Content experts; targeted web searches	2
Nutrition guidelines for schools [34]	Saskatchewan School Boards Association, P/T level NGO	2009	Targeted web searches	1
Breakfast program resource [40]	Alberta Health Services, P/T government organization	2009	Content experts; targeted web searches	2
School meal and school nutrition program handbook [41]	British Columbia Ministries of Education and Healthy Living and Sport, P/T government organization	2010	Custom Google search engines; targeted web searches	2
Moving forward with school nutrition guidelines [27]	Government of Manitoba, P/T government organization	2014	Custom Google search engines; grey literature databases; targeted web searches	3
Healthy eating school resources manual [28]	Newfoundland and Labrador Departments of Education and Health and Community Services, P/T government organization	2006	Targeted web searches	1
Provincial breakfast program standards [29]	Nova Scotia Department of Education, P/T government organization	2007	Content experts; targeted web searches	2
Student nutrition program—nutrition guidelines [30]	Ontario Ministry of Children and Youth Services (MCYS), P/T government organization	2008	Content experts; Custom Google search engines; targeted web searches	3
Ontario’s student nutrition program guidelines [31]	Ontario MCYS, P/T government organization	2014	Content experts; targeted web searches	

**Fig. 2:**
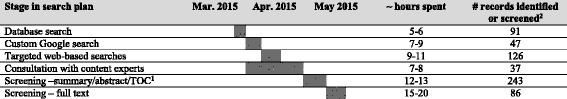
Search plan timeline. This figure demonstrates the timeline and number of hours spent during each stage of the study plan

The search strategies for identifying and screening publications for inclusion in the case study review were found to be manageable, comprehensive, and intuitive when applied in practice. All components of the methodological search plan were conducted in a 2-month period by the lead author (as shown in Fig. 2). The time spent on each phase was approximated retrospectively. The pre-defined search parameters (e.g. reviewing the first ten pages of database findings from each of the ten keyword searches used) enabled a clear and efficient timeframe for conducting the search plan. The search methods yielded publications from a wide range of organizations (i.e. in terms of geographical area, type, and size). Although several publications (11/15 or 73.3 % of the included publications) were found in duplicate following the four identification strategies (often publications highlighted by content experts), there was only a moderate level of overlap in the types of publications these strategies identified. The targeted web searches were the most comprehensive means of identifying relevant publications; this strategy identified all but one of the included publications (as shown in Table 2) [32]. Only one publication was identified through search of grey literature databases; the results of these database searches yielded mostly position papers, program evaluation reports, and other primary research studies [27]. Four of the 15 publications included in the review synthesis were uniquely identified through one strategy [28, 32–34], suggesting that reliance on one source would have led to a less than thorough review. Three of these uniquely identified records were published by provincial non-government organizations.

Excel was a convenient and suitable means of record management in the case study review; however, the program did have some limitations. The advantages of using Excel included the option of exporting records from the grey literature databases directly into a spreadsheet, the ability to duplicate individual sheets on spreadsheets (which was useful for separating the different screening phrases), the remove duplicates feature, the ability to insert an extra column in Excel to input screening decisions, and the ‘sort’ function (i.e. used to sort records by screening decision code, organization name). The disadvantages of Excel included the inability to directly export from Google search engines and time spent switching between the web browser and Excel spreadsheet while recording publication information and screening publications.

Eight of the 15 publications (53.3 %) were from non-government organizations [32–39]. Two of the publications were published at the national level by not-for-profit organizations that provide funds and support for breakfast programs across Canada [35, 36]. Seven of the included items (46.7 %) were from provincial government sources [27–31, 40, 41]. No publications or resources were identified from the federal or territorial governments. The provincial governments in Saskatchewan, Quebec, New Brunswick, and Prince Edward Island were also not represented in the included publications. The publications were released between 2009 and 2015.

The guidelines offered various rationales for the importance of school-based breakfast programs, including creating a leadership opportunity for youth [28, 29, 36], improving school and community connectedness [28, 29, 36, 41], addressing food insecurity [32, 36, 41], reducing the prevalence of obesity in youth [28, 32, 34, 36, 38], providing nutrition education [27–29, 35–37], and encouraging long-term healthy eating habits [31, 37]. All of the documents highlighted how breakfast influences learning outcomes, educational attainment, and healthy development. Most of the guidelines acknowledged the school environment’s unique influence on the youth’s attitudes and behaviours and strived to make ‘the healthy choice the easy choice’ for youth.

## Discussion

This paper presents a unique and explicit method for applying systematic review search strategies to the grey literature. This method was used in a case study to identify 15 publications from provincial government organizations and national and provincial non-government organizations that provided guidance on the delivery of school breakfast programs. If only academic sources of literature were relied upon to identify relevant publications for this case study review, it is likely that only guidelines identified through consultation with content experts or hand-searching journal articles’ reference lists would be included. Within this case study, content experts identified nine of the 15 included publications. As such, nearly half of the included items would have not have surfaced without the use of the other strategies. Grey literature was the central source of information for the subject and publication type needed to conduct the case study systematic review.

The systematic review search strategy described in this article can serve as a template for subsequent grey literature research syntheses, or at the very least serve as a starting point for refining such methods. The search strategy we developed and tested is amenable to adaptation to identify other types of grey literature from other disciplines and answering a wide range of research questions. For example, for step one of the identification strategy, discipline-specific grey literature databases can be searched. A research group interested in identifying reports on climate change can consult environmental grey literature databases (e.g. the University of Texas’ Environmental Policy Collection) [42]. Researchers may find custom Google Search engines in their discipline (e.g. SearchingRadiology.com) [43] or can choose to create their own using the free Google Custom Search platform [44]. Researchers are encouraged to incorporate the methodological search plan outlined in this paper in their review syntheses, whether the syntheses be focused solely on the grey literature or used in parallel with a search of the peer-reviewed literature base. This will ensure transparent and reproducible search methods, a requirement of any strong systematic review.

There are some challenges with methods for systematic reviews of the grey literature, as exemplified by the case study. The very nature of grey literature searches may have introduced a degree of bias in the search results. Web-based searches may introduce bias due to the personalized search features of many search engines, also known as the ‘filter bubble’, which may favour items within a certain geographical or topic area, given the automated relevance rating schemes used in search engines. The case study revealed that there appear to be some limitations with item retrieval through searches of the custom Google search engines. Some searches within these engines report yielding several thousand hits, yet only display a small number of pages of hits. In the systematic review process, it is recommended that two different people screen publications for eligibility. Often, this can be problematic due to the formatting of grey literature (i.e. due to lack of consistent titles and abstracts for each document, lack of capability of Google search engine for automatic uploading and/or exporting of records into a bibliographic software system/Excel). Further, due to the more transient nature of documents published on the Internet, a lack of archiving and the changing nature of website domains/URLs/web site addresses, documents which were available at one time may disappear over time. This makes it difficult for others to reproduce the search results to verify the search strategies or update the review at a later date. Indeed, the fact that one reviewer screened the results in the case study review represents a limitation. The time spent on each aspect of the grey literature search plan (e.g. due to downloading, screening, record management) was not recorded in detail prospectively. The time periods indicated in Fig. 2 were approximated following completion of the case study, representing a limitation to this methods paper. In the case study review, we found that grey literature often contains titles that do not accurately describe the document and are ‘catchy’ but misleading. This presents an additional challenge during title screening. Some publications included in the case study review were missing information or contained ambiguous information on certain publication characteristics (e.g. date of publication, contributors), which posed a challenge during data abstraction. This information is generally explicit on publications in peer-reviewed academic journals and books, likely due to reporting standards and the inclusion of peer-reviewers and editors [6]. However, these details can often be located through other means, such as contacting the organization directly or searching their websites and other publications for references to the item with missing information. These strategies were effective in the case study review. Lastly, standard strength of evidence criteria may not be applicable to certain reviews that contain grey literature. This factor may require researchers to identify or develop different criteria than what are used in other reviews. In spite of these challenges, grey literature search methods should be held to the same rigour, as much as possible, as the systematic review search methods for academic, peer-reviewed literature.

The results of the case study systematic review that are presented in this paper reveal some important findings that would not have been apparent without a comprehensive search of the grey literature. First, no federal or territorial government guidelines were uncovered. The lack of federal government guidelines is unsurprising, given the lack of funding or standards related to school nutrition programs from this level of government in Canada. Numerous organizations have advocated for a national school meal program in Canada [45–48]. The absence of guidelines from the territories is concerning, given the high prevalence of food insecurity and proportion of youth in the population [48–51]. The Healthy Kids Panel recently recommended a universal school nutrition program for First Nations communities and all Ontario publicly funded elementary and secondary schools in a report to the Ontario Ministry of Health and Long-term Care [52]. The advocacy work related to school nutrition programs may be generating progress; Nunavut pledged to develop a working group in 2015 to devise school food guidelines for the territory [50]. The current absence of guidelines from the federal and territorial governments represents a major limitation to the resources available for schools seeking to deliver a school-based breakfast program. Second, this review revealed that breakfast programs and other foods that are offered for free within schools are not captured within many provincial guidelines. For example, provincial school nutrition policies in Ontario and British Columbia explicitly state that they only apply to food and beverages that are sold in schools (e.g. through cafeterias, canteens, or fundraisers). This finding demonstrates the lack of guidelines for breakfast programs within existing provincial school nutrition policies.

## Conclusions

The grey literature is an important source of information for review syntheses. There are some challenges with applying systematic search methods to the grey literature, due to a lack of standards and resources for how to complete these searches and a number of characteristics of the grey literature. This article demonstrated a feasible and seemingly robust method for applying systematic search strategies to identify web-based resources in the grey literature. This method should be further adapted and tested in future research syntheses.

## Additional files

Additional file 1:
**Search results – Custom Google search engines.** This table depicts the ten unique searches applied to the custom Google search engines examined, the number of results each search yielded within each search engine, and the number of records identified from each search. Additional file 2:
**Search results – Targeted web searches.** This table depicts the ten unique searches applied to Google search engine and the number of records identified from each search. Additional file 3:
**Websites identified through targeted web searches.** This table lists the name and link of each website identified through targeted web searches.

## Abbreviations

PRISMA: Preferred Reporting Items for Systematic Reviews and Meta-Analyses
