# Supplementary material for: Applying systematic review search methods to the grey literature: a case study examining guidelines for school-based breakfast programs in Canada
Source: Syst Rev. 2015 Oct 22;4:138. doi: 10.1186/s13643-015-0125-0 (PMC4619264; doi:10.1186/s13643-015-0125-0)
Supplement: Additional file 1: — Search results – Custom Google search engines. This table depicts the ten unique searches applied to the custom Google search engines examined, the number of results each search yielded within each search engine, and the number of records identified from each search. [file 13643_2015_125_MOESM1_ESM.docx]

# Additional File 1. Search results – Custom Google search engines

Custom Google Search Engine for Government Documents

Note: filter was applied to limit search to Canadian documents.

Date searched: 30-Mar-15

| **#** | **Search** | **# results** | **# results screened** | **# new potentially relevant records** | **Total # records** |
| --- | --- | --- | --- | --- | --- |
| 1 | school AND breakfast meal snack milk AND guidelines OR policies | ~ 476,000 | 100 | 18 | 18 |
| 2 | meals OR nutrition OR feeding AND school AND programs | ~ 42,200,000 | 100 | 3 | 21 |
| 3 | school nutrition AND policies or guidelines | ~ 4,210,000 | 100 | 4 | 25 |
| 4 | school meals AND best practices | ~ 4,000,000 | 100 | 0 | 25 |
| 5 | nutrition guidelines AND school program | ~ 3,700,000 | 100 | 0 | 25 |
| 6 | breakfast AND school AND recommendations or guidelines | ~ 4,080,000 | 100 | 5 | 30 |
| 7 | milk program AND school | ~ 7,700,000 | 100 | 2 | 32 |
| 8 | fruits vegetables AND school AND program OR initiative | ~ 2,790,000 | 100 | 3 | 35 |
| 9 | school AND meal OR breakfast AND guidelines OR policies | ~ 13,700,000 | 100 | 0 | 35 |
| 10 | meal program AND school | ~ 22,100,000 | 100 | 0 | 35 |

Searches “All results” – first 10 pages, representing 1000 results screened

Custom Google Search Engine for Canadian Public Health Information

Date searched: 31-Mar-15

Searches “All results” – first 10 pages, representing 1000 results screened

| **#** | **Search** | **# results** | **# results screened** | **# new potentially relevant records** | **Total # records** |
| --- | --- | --- | --- | --- | --- |
| 1 | school AND breakfast meal snack milk AND guidelines OR policies | ~ 466,000 | 100 | 4 | 39 |
| 2 | meals OR nutrition OR feeding AND school AND programs | ~ 36,700,000 | 100 | 2 | 41 |
| 3 | school nutrition AND policies or guidelines | ~ 4,180,000 | 100 | 2 | 43 |
| 4 | school meals AND best practices | ~ 3,850,000 | 100 | 0 | 43 |
| 5 | nutrition guidelines AND school program | ~ 3,690,000 | 100 | 2 | 45 |
| 6 | breakfast AND school AND recommendations or guidelines | ~ 4,850,000 | 100 | 0 | 45 |
| 7 | milk program AND school | ~ 7,730,000 | 100 | 1 | 46 |
| 8 | fruits vegetables AND school AND program OR initiative | ~ 2,520,000 | 100 | 0 | 46 |
| 9 | school AND meal OR breakfast AND guidelines OR policies | ~ 16,100,000 | 100 | 1 | 47 |
| 10 | meal program AND school | ~ 21,100,000 | 100 | 0 | 47 |
