# Supplementary material for: Applying systematic review search methods to the grey literature: a case study examining guidelines for school-based breakfast programs in Canada
Source: Syst Rev. 2015 Oct 22;4:138. doi: 10.1186/s13643-015-0125-0 (PMC4619264; doi:10.1186/s13643-015-0125-0)
Supplement: Additional file 2: — Search results – Targeted web searches. This table depicts the ten unique searches applied to Google search engine and the number of records identified from each search. [file 13643_2015_125_MOESM2_ESM.docx]

# Additional File 2. Search results – Targeted web searches

Google Search Engine (with filter applied to only capture Canadian websites)

Date searched: March 31, 2015

Searches “All results” – first 10 pages, representing 1000 results screened

| **#** | **Search** | **# new potentially relevant records** | **Total # records** |
| --- | --- | --- | --- |
| 1 | school AND breakfast meal snack milk AND guidelines OR policies | 25 | 25 |
| 2 | meals OR nutrition OR feeding AND school AND programs | 7 | 32 |
| 3 | school nutrition AND policies or guidelines | 15 | 47 |
| 4 | school meals AND best practices | 8 | 55 |
| 5 | nutrition guidelines AND school program | 5 | 60 |
| 6 | breakfast AND school AND recommendations or guidelines | 1 | 61 |
| 7 | milk program AND school | 8 | 69 |
| 8 | fruits vegetables AND school AND program OR initiative | 5 | 74 |
| 9 | school AND meal OR breakfast AND guidelines OR policies | 1 | 75 |
| 10 | meal program AND school | 2 | 77 |
