# Supplementary material for: Applying systematic review search methods to the grey literature: a case study examining guidelines for school-based breakfast programs in Canada
Source: Syst Rev. 2015 Oct 22;4:138. doi: 10.1186/s13643-015-0125-0 (PMC4619264; doi:10.1186/s13643-015-0125-0)
Supplement: Additional file 3: — Websites identified through targeted web searches. This table lists the name and link of each website identified through targeted web searches. [file 13643_2015_125_MOESM3_ESM.docx]

**Additional File 3.** Websites identified through targeted web searches

| **#** | **Website name/organization** | **Link** |
| --- | --- | --- |
| 1 | Ontario Ministry of Children and Youth Services | <http://www.children.gov.on.ca/htdocs/English/index.aspx> |
| 2 | Government of Nova Scotia | <http://novascotia.ca/> |
| 3 | Heart & Stroke Foundation | <http://www.heartandstroke.sk.ca/> |
| 4 | Capital Health | <http://www.cdha.nshealth.ca/> |
| 5 | Saskatchewan School Boards Association | <http://www.saskschoolboards.ca/> |
| 6 | Government of Saskatchewan | <http://www.education.gov.sk.ca/> |
| 7 | Government of Manitoba | <http://www.gov.mb.ca/> |
| 8 | Newfoundland & Labrador Department of Education and Early Childhood Development | <http://www.ed.gov.nl.ca/edu/> |
| 9 | Food Secure Canada | http://www.foodsecurecanada.org |
| 10 | Nova Scotia Education & Early Childhood Development | <http://www.ednet.ns.ca/> |
| 11 | Government of British Columbia | <http://www2.gov.bc.ca/> |
| 12 | Student Nutrition Program Central East | <http://www.studentnutritionprogram.ca/> |
| 13 | Nutrition Tools for Schools | http://nutritiontoolsforschools.ca/ |
| 14 | PEI Healthy Eating Alliance | <http://www.healthyeatingpei.ca/> |
| 15 | Cancer Care Ontario | <https://www.cancercare.on.ca/> |
| 16 | Newfoundland and Labrador Teachers' Association | <https://www.nlta.nl.ca/homeq> |
| 17 | Breakfast for Learning | <http://www.breakfastforlearning.ca/> |
| 18 | Government of New Brunswick | <http://www2.gnb.ca/content/gnb/en.html> |
| 19 | Alberta Health Services | <http://www.albertahealthservices.ca/> |
| 20 | Alberta Coalition for Healthy School Communities | <http://www.achsc.org/> |
| 21 | Ever Active Schools | http://www.everactive.org/ |
| 22 | Living Health Schools | http://www.livinghealthyschools.com/ |
| 23 | Healthy Eating at Schools | http://healthyeatingatschool.ca/ |
| 24 | Nutrition for Learning | http://www.nutritionforlearning.ca/ |
| 25 | Healthy Schools BC | <http://healthyschoolsbc.ca/> |
| 26 | Ontario Student Nutrition Program | https://www.osnp.ca/ |
| 27 | Center for Science in Public Interest | http://www.cspinet.org/ |
| 28 | E4C | http://e4calberta.org/ |
| 29 | BC School Fruit and Vegetable Nutritional Program | http://sfvnp.ca/ |
| 30 | Plan Canada | http://plancanada.ca/ |
| 31 | Child Nutrition Council of Manitoba | http://childnutritioncouncil.com/ |
| 32 | Canadian Feed the Children | http://www.canadianfeedthechildren.ca/ |
| 33 | Dieticians of Canada | http://www.dietitians.ca/ |
| 34 | Ontario Ministry of Education | http://www.edu.gov.on.ca/eng/ |
| 35 | British Columbia Ministry of Education | http://www2.gov.bc.ca/gov/theme.page?id=F5415B90D214B10A6245D30D397A05A1 |
| 36 | Apple Schools | http://www.appleschools.ca/ |
| 37 | Healthy Alberta | http://www.healthyalberta.com/ |
| 38 | HealthLink BC | http://www.healthlinkbc.ca/ |
| 39 | EatRight Ontario | http://www.eatrightontario.ca/en/default.aspx |
| 40 | Health Canada | http://www.hc-sc.gc.ca/index-eng.php |
| 41 | Government of Prince Edward Island | http://www.gov.pe.ca/ |
| 42 | Institut National de Sante Publique Quebec | http://www.inspq.qc.ca/english/ |
| 43 | Alberta Department of Education | https://education.alberta.ca/ |
| 44 | Ontario Society of Nutrition Professionals in Public Health | http://www.osnpph.on.ca/ |
| 45 | Physical & Health Education Canada | http://www.phecanada.ca/ |
| 46 | Legislative Assembly of Alberta | http://www.assembly.ab.ca/ |
| 47 | Nourish Nova Scotia | https://nourishns.ca |
| 48 | Canadian Best Practices Portal | http://cbpp-pcpe.phac-aspc.gc.ca/ |
| 49 | Childhood Obesity Foundation | http://www.childhoodobesityfoundation.ca/ |
| 50 | Feed Tomorrow | http://feedtomorrow.ca/ |
| 51 | Northwest Territories | http://news.exec.gov.nt.ca/ |
| 52 | Public Health Association of BC | http://www.phabc.org/ |
| 53 | Action Schools! BC | http://www.actionschoolsbc.ca/ |
| 54 | Food Security Network of Newfoundland & Labrador | http://www.foodsecuritynews.com/ |
| 55 | Provincial Health Services Authority | http://www.phsa.ca/ |
| 56 | Student Nutrition Program of Central Eastern Ontario | http://www.studentnutritionprogram.ca/ |
| 57 | Investing in Children | http://investinginchildren.on.ca/ |
| 58 | Pan-Canadian Public Health Network | http://www.phn-rsp.ca/index-eng.php |
| 59 | Mavalicious KidsEat | http://www.mavaliciouskidseat.com/about |
| 60 | Thought About Food? | http://www.foodthoughtful.ca/ |
| 61 | First Nations Health Council | http://fnhc.ca/ |
| 62 | Dairy Farmers of Canada | http://www.dairyfarmers.ca/ |
| 63 | Dairy Farmers of Ontario | https://www.milk.org |
| 64 | Dairy Farmers of Manitoba | https://www.milk.mb.ca/ |
| 65 | Saskmilk | http://www.saskmilk.ca/ |
| 66 | Ophea Healthy Schools Healthy Communities | http://www.ophea.net/ |
| 67 | More about Milk | http://www.moreaboutmilk.com/ |
| 68 | PEI School Milk Foundation | http://peischoolmilk.ca/ |
| 69 | Chep Good Food Inc. | http://www.chep.org/en |
| 70 | Food and Farming Canada | http://www.foodandfarmingcanada.com/ |
| 71 | British Columbia Dairy Association | http://bcdairy.ca/ |
| 72 | Government of Ontario | https://www.ontario.ca/ontario-government |
| 73 | Ontario Ministry of Healthy & Long-term Care | http://www.health.gov.on.ca/en/ |
| 74 | Agriculture in the C-lassroom Canada | http://www.aitc-canada.ca/en/ |
| 75 | Breakfast Club of Canada | http://www.breakfastclubcanada.org/ |
| 76 | Food First Foundation | http://foodfirst.ca/ |
| 77 | Ontario Edible Education Network | http://sustainontario.com/ |
